# Understanding the conditions for inclusive education: A realist evaluation of a French territorial innovation

**DOI:** 10.1371/journal.pone.0348203

**Published:** 2026-04-29

**Authors:** Eléonore Ségard, Olivier Aromatario, Philippe Chervin, Linda Cambon

**Affiliations:** 1 International Foundation of Applied Disability Research (FIRAH), Paris, France; 2 Team EVIDANS, MeRISP, Centre Inserm U1219 de l’Université de Bordeaux, CHU de Bordeaux, Bordeaux, France; Father Muller Charitable Institutions, INDIA

## Abstract

Article 24 of the United Nations Convention on the Rights of Persons with Disabilities (UNCRPD) calls for inclusive education systems. Despite this mandate, collaboration between schools and specialized services often remains limited. This study examined the systemic conditions that enable effective inclusion, extending beyond pedagogical approaches through a complexity-informed framework. Using a realist evaluation, the research investigated an innovative inclusive education initiative implemented in the Eure-et-Loir district in France for children and young people with neurodevelopmental disabilities (CYWD). The study sought to identify the mechanisms that foster autonomy and social participation among CYWD and to determine the conditions under which these mechanisms emerge. Two cases—one in a primary school and one in a secondary school—were analyzed. In total, 47 semi-structured interviews were conducted with 63 participants (CYWD, classmates, families, specialized professionals, national education staff, and school life personnel). The analysis identified 162 ingredients across four domains: the district-level inclusive education scheme, the National Education system, the Specialized system, and their interinstitutional cooperation. These ingredients were combined into 15 success conditions that activate five key mechanisms within CYWD: developing a sense of safety and stability, feeling fully part of the class, recognizing and using accommodations, motivation to learn, and motivation to engage socially. These mechanisms contributed to learning progress (often developing in unexpected ways), greater autonomy, increased initiative, and richer peer participation, including outside the classroom. Broader systemic benefits were also observed, including a general climate of kindness in schools and increased empowerment among families. The study demonstrates that high-commitment inclusive education practices can be implemented at a district level and are perceived to have a positive impact on CYWD. It identifies key systemic and collaborative configurations that can guide the development and transferability of effective inclusive education practices across diverse contexts.

## Introduction

### Inclusive education

Inclusive education has become a central principle in international educational policy, particularly since the adoption of the United Nations Convention on the Rights of Persons with Disabilities (UNCRPD). The UNCRPD reflects a shift from a medical model, which locates disability within the individual, to an interactive model that views disability as arising from the interplay between personal characteristics and environmental conditions [1]. This perspective moves the focus away from “fixing” the person and toward improving the fit between individual capacities and strengths, and the demands of the contexts in which people live, learn, work, and play. It promotes a support-based model in which settings are made more accessible and human service systems provide adequate assistance, placing responsibility on environments and service providers rather than on individuals to adapt.

Article 24 of the UNCRPD affirms the equal rights of persons with disabilities to participate in mainstream education at all levels and to access lifelong learning. In line with this, the United Nations Children’s Fund (UNICEF) defines inclusive education as a system that welcomes and supports all students to learn together in the same schools, regardless of their abilities or needs. Despite this international consensus, many children with disabilities remain excluded from education or are placed in segregated settings. Inclusive education is particularly challenging for children with cognitive, behavioral, and intellectual disabilities compared with children who have physical disabilities or sensory impairments [2].

### Barriers and challenges

Implementing inclusive education continues to face persistent barriers, including insufficient resources, negative societal attitudes, limited teacher training, inaccessible infrastructure, and standardized assessments that fail to accommodate diversity [3–7]. Several challenges have been identified. Transitioning from special education to inclusive education requires deep structural transformations across both educational and specialized systems. This shift entails redefining the role of special schools from providers of segregated education to partners and resource centers for mainstream schools [8,9] and strengthening collaboration between general education staff and specialized service providers [10]. It involves reorganizing institutional practices and improving coordination among diverse stakeholders, including teachers, therapists, support workers, medical professionals, and local authorities [11–13]. Although collaborative action is widely recognized as a cornerstone of inclusive education, its practical implementation and dynamics remain underexplored [14]. Other challenges include the need for systemic changes at all levels and the broader transformation of competitive, individualistic societies into inclusive ones—a paradoxical and complex task [15,16].

Without careful planning, inclusive policies risk resulting in the mere physical presence of children and young people with disabilities (CYWD) rather than ensuring their quality of experience and genuine participation, particularly for students with cognitive and behavioral disabilities. This is mainly because their needs are more complex and variable and because the implementation of necessary support tends to be less consistent.

### Transferability of inclusive practices

While local initiatives often yield valuable insights, they frequently remain fragmented and struggle to achieve broader dissemination. The transferability of successful disability-related practices is constrained by contextual variability, complexity, and the lack of systematic adaptation frameworks [17]. Nevertheless, innovative approaches can generate evidence-based, transferable knowledge and practical tools to support the transition toward inclusive education—provided they address real needs and ensure applicability across contexts.

Bridging ideological commitments with empirical evidence is essential. Current research lacks clarity on which approaches and configurations are most effective in sustaining social inclusion, and there are limited data on the essential characteristics of inclusive school models and inclusive education practices [2]. Research should focus on the pressing question: “How do we operationalize inclusive education?” [18]. Capacity-building and identifying local conditions for success are crucial. Recent work on the transferability of social innovations in the disability field has called for theory-driven approaches that move beyond listing enabling factors, to more fully elucidate the mechanisms underlying change and their interactions with context [19]. Mechanisms are viewed as pivotal functions that must be replicated to ensure successful transferability [19]. Understanding those mechanisms, as well as the conditions that activate them, helps in the determination of how to adjust an intervention to fit each new context, thereby supporting the emergence of expected mechanisms. In addition, the Medical Research Council (MRC) recommends theory-driven evaluation for this type of complex intervention, as it involves several components that can interact with each other and whose effect is the result of interaction with the context in which it is implemented [20]. The evaluation of a complex intervention cannot be limited to an evaluation of results and requires an understanding of the processes and mechanisms at work.

Thus realist evaluation offers a suitable framework to address transferability by integrating this complexity and focusing on how, why, and in what contexts interventions produce their effects. A 2019 realist review contributed to the field by identifying contextual conditions from a systemic perspective, but it was limited by its literature-based scope and descriptive aims. It did not examine in detail how specific conditions combine to enable effective inclusion of CYWD [21].

### Study rationale and aim

The present study applies a realist evaluation to an innovative inclusive education initiative in France targeting children and young people with neurodevelopmental disabilities. By analyzing its territorial, systemic, and collaborative dimensions, this research aimed to identify key contextual and interventional components and to understand how specific configurations shape outcomes through mechanisms [22] for school-aged children and young people with disabilities. The goal was to inform the development, transferability and scaling of effective inclusive education practices in complex and variable settings.

## What this paper adds

- Identifies five key mechanisms that underpin effective inclusive education. Using a realist evaluation, the study specifies how safety, belonging, recognition of difference, and motivation processes generate learning, autonomy, and participation outcomes for students with neurodevelopmental disabilities.
- Moves beyond barriers and facilitators to explain how inclusion works., this study links concrete contextual configurations to outcomes through explicit Context–Mechanism–Outcome explanations.
- Demonstrates that effective inclusion depends on a coordinated systemic configuration. By mapping 162 interventional and contextual ingredients across governance, mainstream education, specialized services, and cooperation, the study shows that inclusion requires structured inter-institutional alignment—not only classroom adaptations and pedagogical techniques. This systemic mapping contributes detailed empirical insight into how population-level responsibility for inclusion can be operationalized within a defined territory.
- Provides a transferable framework for action. Rather than proposing a fixed model, the study identifies 15 activation conditions paper offering operational guidance for adapting and scaling inclusive education models in other contexts.
- Expands the scope of outcomes considered in inclusive education research.

In addition to student learning and autonomy, the study documents effects on families, peers, and school climate, supporting a broader understanding of inclusion as a population-level and societal intervention.

## Methods

### Study design and reporting standards

We employed a qualitative design. An embedded case study design was selected because it is particularly well suited to examining the complex interrelationships of a phenomenon within its real-life context and to capturing the diversity of its implementation [23]. Two cases were selected for parallel investigation using a convergent analysis: one located in a primary school and the other in a secondary school.

This study is reported in accordance with the Realist and Meta-narrative Evidence Syntheses: Evolving Standards II (RAMESES II) (S1 File).

### Theorical framework: Realist evaluation

Realist evaluation is aimed at understanding how and why an intervention works in a given context, rather than simply assessing whether it works [24]. It is grounded in the idea that interventions do not produce universal effects, postulating, instead, that outcomes depend on the contexts in which they are implemented. This approach relies on a generative or contingent causal model. Rather than aiming to identify a single cause–effect relationship, as experimental methods (e.g., clinical trials) do, realist evaluation examines how a positive or negative phenomenon emerges, under what conditions it emerges, and through which causal or mechanistic processes. This is particularly useful for analyzing complex systems whose components are numerous, varied, and mutually influential. By exploring different configurations of these components and the effects they produce, realist evaluation allows for circumstantial conclusions and helps identify combinations of factors that promote the emergence of a given effect.

To do this, and like any theory-based evaluation [25], realist evaluation relies on the formulation of middle-range theories that explain the conditions under which a measure does or does not produce a desired effect. These theories take shape as causal hypotheses structured according to the Context–Mechanism–Outcome (CMO) heuristic:

- C (context): Structural, organizational, institutional, and individual conditions in which the intervention is implemented.
- M (mechanism): Processes through which the intervention produces an effect, including the reactions and interactions of those involved.
- O (outcome): Observed effects, which may vary depending on the combinations of context and mechanism.

Realist evaluation typically draws on multiple case studies [19,24,26], selected for their differences and analyzed in parallel, sequentially, or using a hybrid approach. CMOs may be defined at an early stage and then validated, refined, or revised through data collection across cases. Alternatively, using an inductive approach, they can be constructed and validated as the cases are analyzed. Developing robust initial CMOs is often a complex process: existing evaluations, literature, and expert opinion can help identify “areas of focus”, meaning aspects that warrant detailed exploration. These represent hypothetical premises that are refined, adjusted, and expanded throughout the analysis to become CMOs. Realist evaluation, therefore, involves continual movement between theoretical and empirical data, enabling increasingly concrete definitions of CMOs—a process known as retroductive theorizing [27].

### Study context: The Eure-et-Loir inclusive education model

Since 2016, the French district (*département*) of Eure-et-Loir has implemented an innovative territorial approach to inclusive education—also called “school for all” in France—for children with neurodevelopmental disorders. This approach was developed through a coordinated partnership among key actors. The innovation was selected for its maturity and its potential to mainstream disability inclusion while creating a model that could be transferred to other French districts and generate evidence on how to implement inclusive education. It reflects a shift from isolated institutional responses to a territory-wide inclusion model based on population-level responsibility and grounded in inter-institutional collaboration.

In this model, all local actors—education, health, and social services—share collective responsibility for ensuring that every child with a disability in the territory has access to inclusive, high-quality educational opportunities, regardless of their location or level of need.

The strategy is jointly coordinated by:

- The regional health authority (*Agence Régionale de Santé*), responsible for organizing and coordinating support services for people with disabilities.
- The local representation of the Ministry of National Education, in charge of the general education system.
- The county-level public agency (“*Maison Départementale de l’Autonomie”*), which serves as the central access point for rights, support, and services for individuals with disabilities and their families. It is responsible for officially recognizing disability status and granting access to entitlements, including additional support from specialized service providers.
- The network of specialized service providers working in the district to support children with neurodevelopmental disorders.

A sector-based territorialization of service provision has been established to ensure comprehensive coverage across the district. Each sector brings together schools and service providers to encourage proximity-based collaboration. This inclusive framework is anchored in the legal principle that mainstream schooling is the default educational setting for all children. In accordance with Article 19 of the French Law of 11 February, 2005, any student with a disability or disabling health condition should be enrolled in the nearest school (*école*, *collège*, or *lycée*) to their home, which serves as their reference institution.

This system serves students with officially recognized disabilities: those identified by the local public agency as having neurodevelopmental disorders and entitled to additional specialized support. These students can attend classes in mainstream settings. They may receive in-class human support from an assistant (*Accompagnant des Élèves en Situation de Handicap* [AESH]). They also receive tailored support packages designed and delivered by service providers. Such support packages may include in-class assistance from specialized staff (e.g., special education teachers, educators) as well as complementary therapeutic, educational, or pedagogical interventions provided on school premises, in nearby facilities, or within the service provider’s structure. This territorial configuration upholds students’ right to inclusive education while ensuring continuity of care and access to specialized support when needed.

As part of this transition, traditional specialized institutions (*Institut Médico-Éducatif* [IME]) have undergone a major transformation into new structures (*Dispositif d’Accompagnement Médico-Éducatif* [DAME]), enabling them to function as flexible, mobile, school-facing support platforms rather than segregated institutions. DAME are designed to support fully the social and educational inclusion of children and youth with disabilities while providing concrete resources and expertise to school teams to facilitate inclusive practices [28].

The Eure-et-Loir model emphasizes not only academic achievement but also the emotional and social development of learners at school. Moreover, the inclusion project extends beyond school to encompass the broader social inclusion of children and young people with disabilities within their living environments—family, extracurricular activities, neighborhood life, and more. In practice, this territorial approach has also been applied to leisure centers through the creation of a dedicated district-level disability resource team.

### Realist evaluation phases

The study aimed to identify the mechanisms (M) that underpin an inclusive school environment promoting autonomy and social participation of CYWD (O) and to explore the conditions under which these mechanisms emerge (C) within the initiative implemented in the Eure-et-Loir district.

#### Phase 1: Define the CMOs.

Although existing literature addresses the bases of disability-based discrimination and exclusion and proposes constructs to conceptualize the experience of people with disabilities (e.g., [1,29–32]), studies often lack detailed analysis—consistent with realist evaluation—of the observable structural processes within specific contexts that produce such exclusion. Given the absence of sufficiently fine-grained and operational initial middle-range theories that we could seek to validate, we opted to conduct our study using an inductive model. At this stage, therefore, we did not have initial stabilized CMOs to test but, rather, an analytical framework grounded in how we defined C, M, and O in our context.

A mechanism (M) was defined as a perception, a response, or a skill demonstrated by school-enrolled children or young people receiving support from specialized services. Expected mechanisms were based on the principles of the UN Convention on the Rights of Persons with Disabilities. These principles were translated into potential mechanisms (e.g., feeling a sense of belonging to the community, feeling valued) (see S1 Table). Outcomes (O) were defined as situations of social participation of children and youth with disabilities, both in school and in community life. We also examined effects on families and on children without disabilities.

We distinguished two types of Contexts (C):

- An interventional ingredient (Ci), defined as an element internal to the inclusive school model as implemented in Eure-et-Loir and one over which local stakeholders (e.g., the Regional Health Agency, the district’s Inclusive School Service, specialized services, schools, and professionals from these institutions) can exert influence.
- A contextual ingredient (Ce), defined as a condition arising from the external environment of the inclusive school model and one that cannot be modified by local actors. Examples include national education policies, teacher reassignment dynamics, or pre-existing partnerships between territorial actors.

#### Phase 2: Identify and refine the CMOs.

The aim of this phase was to collect empirical data documenting the presence or absence of each component—Ce, Ci, M, and O—and to assess the relationships between these elements using a configurational approach.

Data collection: This study focused on CYWD and included in-depth interviews with the students themselves as well as with individuals in close interaction with them. To explore district-level organization, we also interviewed the person responsible for inclusive education at the Regional Health Agency, along with the director and a coordinator from the Inclusive Education Service within the district’s National Education system. Inclusion criteria were the following: i. for CYWD: be under 15 years of age, have a recognized disability status by the relevant public authority, be entitled for support from a specialized service provider (DAME), be enrolled in one of the schools included in the study ii. for children without disabilities: be under 15 years of age, be in the same class as a child with a disability who is already participating in the study iii. for families: father and/or mother holding parental authority over a child with a disability participating in the study, aged over 18 years, not under guardianship or curatorship iv. for professionals: working in one of the two schools selected for the research. The recruitment process took place in April and May 2024. In each school, CYPD who met the selection criteria were identified. The study was presented to them and their families by the DAME educational coordinator. Subsequently, the study was proposed to children in the same classes and to the professionals working with these CYWD. This was done by the management teams of the DAME institutions (for DAME professionals) and by the school administrations (for families of children without disabilities and National Education professionals). An information sheet and a joint letter from the Regional Health Agency and the National Education authority describing the study were distributed to all families and professionals. In addition, two videoconference sessions were organized by the research team to present the study to potential participants.

Interviews took place from the 3^rd^ of June 2024 to the 13^th^ of July 2024. We used semi-structured interviews. Examining participants’ accounts enabled us to identify Ce, Ci, M, and O, as well as the relationships they perceived among these elements within the context of inclusive education. An interview guide, tailored to each group (S2 File), explored the following dimensions: i) attitudes of CYWD toward school and learning, school organization and accommodations, social relationships and school life, school pathways and future plans, and life outside school ii) the evolution of professional practices in both National Education and specialized services iii) the ways in which partnerships between education and specialized sectors were implemented iv) contributions from the territorial dynamic supporting this transformation.

Consistent with the realist approach, we left open the possibility for other elements to emerge. Interviews with adults lasted approximately 1 hour, were conducted face-to-face, and were audio-recorded and transcribed. Group interviews were conducted with classmates and were not audio-recorded; the researcher took detailed notes during and immediately after each session. Interviews with CYWD were conducted either individually or in small groups (2–3 children), with the format determined in consultation with specialized professionals familiar with each child. The goal was to create conditions in which students felt comfortable. In some cases, individual interviews were preferred (e.g., to avoid emotional contagion, mimicry, or distractibility); in others, group settings were chosen (e.g., to avoid singling out a child or to foster more dynamic interaction).

Because the field of inclusive education rarely incorporates the perspectives of the primary participants—students with disabilities themselves [33,34]—and because some students were non-verbal, adapting interviews with CYWD to ensure accessibility and meaningful participation was central to the study. Preparation involved co-designing clear, simple interview guides with specialists, using visual and emotional aids, and creating familiar, low-stimulation environments. During interviews, adaptations addressed communication, attention, and fatigue challenges through flexible pacing and supportive tools. Interpretation was strengthened through debriefings with psychologists and teachers, whose contextual insights and cross-validation with other data sources supported accurate, holistic understanding of each child’s responses. The full list of adaptations is presented in S2 Table.

### Ethics and consent

All methods used in this study were conducted in accordance with relevant guidelines and regulations. The study complies with the French MR004 reference methodology. The institutional review board of the Hospital of Bordeaux approved the study protocol the 24^th^ of April 2024 (CER-BDX 2024–42). Written informed consent was obtained from all adults, and written parental consent was obtained for CYWD. Students without disabilities were interviewed on a parental non-opposition basis. Data were anonymized to preserve participant confidentiality.

### Data analysis

We sought to identify and synthesize evidence demonstrating which mechanisms were important in generating autonomy and participation outcomes, and to determine which aspects of the inclusive education model and broader context shaped these mechanisms. Two successive analyses were conducted on each unit of analysis using NVivo® 14 software.

First, a thematic analysis was conducted to identify mechanisms, contextual elements and outcomes reported by participants. Coding focused on elements related to Ce, Ci, M and O emerging from the interview data. In a second stage, a configurational analysis was undertaken to reconstruct context–mechanism-outcome relationships. This interpretive step was informed by principles of phenomenological analysis to examine how participants experienced and interpreted the intervention within specific contextual conditions [35]. Stakeholder triangulation was used to enhance analytic rigor by incorporating perspectives from CYWD, families, professionals, and institutional representatives.. Regarding coding procedures, all interview data were coded using a structured coding scheme based on the intervention system logic. We revisited the entire corpus of interviews for each mechanism, systematically linking coded data to the corresponding context elements (Ci and Ce). Importantly, no data were excluded from the analysis, in order to avoid selective interpretation and to maintain analytical completeness. The interpretation remained closely grounded in the empirical material: associations between context elements and mechanisms were retained only when they were clearly expressed or strongly supported in participants’ accounts. Coding was conducted iteratively, with regular team discussions (ES, OA, LC) to refine CMO configurations. Emerging CMO configurations were compared across the two case sites to identify demi-regularities consistent with realist evaluation approaches [36,37].

## Results

Participants included 10 CYWD, 16 classmates, 5 members of families, 13 specialized professionals, 12 national education professionals, 4 school life staff members (see S3 Table for the breakdown of interview participants in both settings). A total of 47 semi-structured interviews were conducted with 63 participants, individually or in groups in the case of the children.

Results are presented by categories C, M, and O, followed by a section outlining the conditions for activating mechanisms.

### Interventional and contextual ingredients (Ci and Ce)

In total, 162 ingredients (138 Ci, 24 Ce) were identified and categorized into three domains according to their affiliation (inclusive school scheme as implemented at the district level, National Education, specialized system, and cooperation between National Education and the specialized system). Within each category, ingredients were further grouped into sub-domains. The following sections present the categories and sub-domains with examples of ingredients. The full list of ingredients is provided in S3 File.

#### Ingredients pertaining to the inclusive school scheme as implemented at the district level (n = 22, CiED1–CiED22).

Ingredients linked to schooling modalities (CiED1–15), such as CYWD being enrolled in their local school (CiED1).

##### Ingredients linked to the transformation into DAME (CiED16–17):

such as the sectorization of DAME intervention areas (CiED16).

***Ingredients linked to partnerships and resources (CiED18–22):*** such as partnerships with local authorities (CiED19).

Ingredients pertaining to DAME (n = 68; 59 CiDAME, 9 CeDAME).

Ingredients related to the interventions of DAME professionals

1 Ingredients related to supporting CYWD (CiDAME1–8), such as developing a personalized and holistic weekly schedule (CiDAME1)
2 Ingredients related to the resource function with teachers
  i Reassuring teachers about DAME’s approach (CiDAME9–22); for example, being available (CiDAME11)
  ii Demonstrating credibility regarding DAME’s ability to support teachers (CiDAME23–26); for example, providing small and practical solutions (CiDAME24)
  iii Transferring skills to teachers (CiDAME27–32); for example, demonstrating possible adaptations teachers can adopt and make their own (CiDAME28)
  iv Co-constructing with teachers (CiDAME33–35); for example, proposing co-teaching (CiDAME33)
3 Ingredients related to classroom intervention methods (CiDAME36–39), such as being discreet when giving oral instructions so as not to disrupt the class (CiDAME36)
4 Ingredients related to interventions with families (CiDAME40–43), such as guiding families to contact the teacher directly (CiDAME40)

##### Ingredients related to the support system:

1. Structural ingredients (CiDAME44, CeDAME1–2), such as the individual nature of human-assistance compensations granted by the district-level public agency to CYWD (CeDAME1)
2. Organizational ingredients (CiDAME45–49, CeDAME3–4), such as ensuring the availability of sufficient human resources (CiDAME45)
3. Ingredients related to team functioning (CiDAME50–59, CeDAME5–7), such as maintaining a close link between the service manager and the school leadership team (CiDAME50)
4. Ingredients related to partners (CeDAME8), such as the stability of professional partners supporting CYWD

##### Ingredients related to National Education (52 CiEN and 16 CeEN).

**Ingredients concerning conditions for welcoming CYWD into the school and classroom**

1. Ingredients concerning chosen schooling arrangements (CiEN1–2), such as having an appropriate and adapted schooling configuration (CiEN1)
2. Ingredients concerning CYWD’s inclusion in school life (CiEN3–5), such as the possibility to take part in all class and school activities without logistical barriers (CiEN3)
3. Ingredients concerning classroom functioning (CiEN6–15, CeEN1), such as ensuring that the seat next to the CYWD is not reserved for the AESH (CiEN6)
4. Ingredients concerning the teacher’s intervention with CYWD (CiEN16–29), such as time dedicated individually by the teacher to get to know the CYWD and monitor their progress (CiEN16).

##### Ingredients related to the school and the Academy

1. Ingredients concerning learning and training (CiEN30–32), such as training on inclusive education offered to teachers (CiEN29)
2. Ingredients concerning school leadership support (CiEN33–42), such as including quantitative co-teaching objectives in the school project (CiEN36)
3. Ingredients concerning the support of the Academy’s Inclusive Education Service (CiEN43–54), such as offering joint DAME/National Education training sessions (CiEN44)

##### Ingredients related to the national system

1. Structural ingredients (CeEN2–5), such as insufficient time allocated for preparing the school year (CeEN3)
2. Ingredients concerning pedagogical approaches (CeEN6, CiEN52), such as the development of Universal Design for Learning (UDL) (CiEN52)
3. Ingredients concerning teacher management (CeEN7–11), such as staff assignment and transfer procedures within National Education (*le mouvement*) (CeEN8)
4. Ingredients concerning the functioning of Inclusive Education (CeEN12–15), such as shifting policies and the proliferation of experiments that may interfere with locally implemented systems (CeEN13)
5. Ingredients concerning the plurality of programs (e.g., allophone students, gifted students), which remain compartmentalized (CeEN16)

##### Ingredients related to DAME/National Education cooperation (5 CiCoDE).

Five intervention-related ingredients were identified (CiCoDE1–5), such as teamwork between institutions (CiCoDE3).

#### Mechanisms affecting CYWD.

Five mechanisms affecting CYWD were identified.

**Mechanism 1: Understanding the school environment (M1).** In the studied French district, CYWD are enrolled in their local mainstream school, with support from specialized professionals to meet their educational, social, and therapeutic needs. Schooling for CYWD often involves multiple locations, schedules, and professionals, which can create an unstable environment—particularly when associated impairments are present (e.g., disorientation, fear of change, behavioral crises). A key mechanism for successful schooling is fostering a sense of safety and stability. CYWD must be able to develop familiar reference points in a predictable environment, supported by clear routines and coordinated action among all stakeholders.

**Mechanism 2: Feeling like a student (M2).** The student must develop a sense of belonging to the school and their classroom. They should not feel excluded or rejected. To feel like a student “like any other”, the child or young person must experience interactions and attention similar to those of their peers, without being singled out or stigmatized.

**Mechanism 3: Acknowledging one’s own difference (M3).** For CYWD, a successful educational experience involves both belonging within the peer group and acknowledging their specific needs. While it is important that they feel similar to their classmates, it is equally essential that they recognize the distinct nature of their disability. This includes understanding the purpose of support measures and accommodations, actively engaging with them, and perceiving their added value in facilitating learning and participation.

**Mechanism 4: Motivation to engage in learning (M4).** One of the central aims of schooling CYWD is access to learning. However, beyond simple access, effective learning requires that the student not only be able to learn but also genuinely want to engage in learning.

**Mechanism 5: Motivation to engage in social life and ability to project oneself into the future (M5).** Beyond academic learning, immersion in a mainstream environment allows CYWD to invest in their lives as members of society—first as children or teenagers (at school, with family, during leisure activities, within their neighborhood) and later as adults (in professional and civic life).

#### Outcomes.

The study focused primarily on CYWD, and three types of outcomes (O1, O2, O3) were identified. However, outcomes concerning families (O4, O5) and classmates (O6, O7) also emerged from the data. Because these outcomes directly influence CYWD, they are described here as well.

##### Outcomes for CYWD.

**Outcome 1: Developing learning skills (O1).** Several testimonies reflect a sense of surprise at the skills developed by CYWD—sometimes completely unexpectedly. The term “revelation” was mentioned. Often, the mere fact that a CYWD engages in learning “surprises” professionals who did not expect progress to occur so quickly, in a specific subject, or with such magnitude. Schooling helps keep the field of possibilities open and allows the potential of CYWD to emerge. Comments included: “*We discover that, well, they have abilities that are… different, but surprising to us*,” “*It’s full of surprises and it’s constant*,” and “*Now he’s a young person who develops psychosocial skills despite his impairment, in a middle school, quite incredibly*.” Being in school exposes CYWD to a stimulating environment—something that may be less present in specialized settings: “*It’s very important, I think, for brain development*.”

It is not possible to draw up a common list of acquired skills because they depend on each child; the emphasis is on the progress made. Linked to this effect, developing a taste for school also emerged: “*He really enjoys working*.”

**Outcome 2: Acquiring autonomy and developing initiatives (O2).** The results show that CYWD acquire autonomy—“*She now knows how to organize her materials*”—and develop the ability to take on responsibilities and initiatives, whether at school (e.g., speaking in front of the class) or in family life (e.g., cooking pasta).

**Outcome 3: Interacting with peers (O3).** Being enrolled in their local school enables CYWD and other students to encounter one another in shared community spaces (parks, supermarkets, the city center). CYWD may thus develop multiple social anchors across different places: “*When he’s at home in his village, he goes out, plays with his friends, plays basketball*.” This effect is reinforced by participation in the leisure center, made possible through the territorial dynamic.

Interviews also revealed that for some CYWD, such outcomes do not materialize, and inclusion may even cause suffering. Certain signs may indicate that inclusion is not going well. Behavioral changes, for example: “*On top of his severe disabilities—he had trouble walking, talking, etc.—he began to develop OCD. So the mother noticed something was wrong at school*.” Another sign might be a CYWD being alone during recess, though this is not always negative—it may be chosen and serve as a resourceful moment: “*Actually, it’s about whether it’s an isolating bubble or a resourceful moment*.”

Professionals describe how challenging it can be to decide whether to persevere with inclusion or stop in such situations. Decision-making is facilitated by the ability to work as a team—to collaborate around the student with a collective vision, share perspectives, and involve families, “*So that we can really cross our views on the functioning, needs, and expressions of the young person*.” Professionals emphasize that it is not possible to determine in advance whether inclusion will be beneficial; there appear to be no predictive factors.

##### Outcomes for families.

**Outcome 4: Choosing their child’s school path (O4).** Inclusive schooling encourages parents to become active participants in their child’s educational project. Some parents still do not feel legitimate as parents of a school student; they hesitate to interact with teachers or to register their child for leisure centers or even the cafeteria. Systematic schooling helps change this dynamic. When services are offered without requiring “begging” or additional paperwork, access to rights is facilitated, and families are more able to express their specific needs and expectations: “*And I think it becomes a habit. People know it. So now they’re no longer afraid to take the step, to come see us and ask that their child participate in extracurricular activities*.” “*And little by little, now we have families telling us, I want my child to go to school, I want… we’re really in new services, they are able to request services, the parents.”* Furthermore, it has been noted that some families adopt a rights-based posture from the outset.

Continuity in inclusive schooling from primary to secondary school (as allowed in the district’s model) reassures parents in their choices. They feel able to commit to this mode of schooling without fearing a disrupted path. The issue of access to vocational training, however, remains.

**Outcome 5: Being a parent, not just a parent of a child with a disability (O5).** When their child is enrolled in school, parents can view themselves as parents of a student. They are no longer stigmatized by the type of institution their child attends. Families may also tend to overprotect their child—for example, by refusing birthday invitations because the experience is unfamiliar and feels risky. Schooling shows them that their child can manage in a mainstream environment, is making progress, and is gaining autonomy in certain areas. It may help them see their child as capable, believe in their potential, and imagine a future for them. As one professional noted: “*Parents are sometimes very surprised when we tell them [what their child does at school]. (…) Yes, but he is capable. Sometimes it’s good for parents to hear that too*.”

Only one interviewed family shared their child’s career plan (a sheltered workplace) and their vision for his future: “*What I want for him is not to spend his whole life at home. I see a supported living facility, an apartment where he’s independent*.”

##### Outcomes for peers and the school.

**Outcome 6: Developing values of tolerance (O6).** Interviews indicate that students without disabilities are very open, and having a classmate with a disability is not considered remarkable: “*It’s normal that they (students with disabilities) are with us*.” Few instances of mockery were reported. At leisure centers, children appear similarly accustomed: “*They immediately go toward them [CYWD]. They’re really attentive and caring. There’s none of the fear, questions, or meanness you sometimes see between peers in the schoolyard*.”

The presence of CYWD also plays an educational role for their classmates by exposing them to difference and helping them to develop tolerance and engage in dialogue: “*School shows you there are people with disabilities, there are others, and everyone can live together*.” Another student noted: “*In classes where there are no children with disabilities, they may not really understand what it is and therefore have more difficulty adapting or knowing how to react. But students who have disabled classmates—they’re with them every day or half days. I think they get used to it a lot*.”

Interviews with students revealed that they regretted not being informed at the start of the year when a CYWD joined the class. They would have liked to know, for instance, what the student did when not in class, where they went, or how to behave with them. They had questions but did not dare to ask. They emphasized that this information was not essential—but if time and a designated person had been offered to address these questions, they would have welcomed it.

**Outcome 7: A “global” climate of kindness (O7).** This climate of kindness within the school appears to extend to other issues, including bullying.

#### Conditions of activation of mechanisms.

It was not possible, based on the data, to determine whether a given mechanism specifically activates one particular outcome. Rather, the analysis suggested that the five mechanisms (M1–M5) collectively are necessary to produce the seven outcomes described (O1–O7).

By contrast, the data were highly informative regarding the conditions required to activate the mechanisms.

Fifteen success conditions—composed of 55 combinations of 133 contributing ingredients—must be present to activate the first 4 mechanisms. Each condition is presented below. It is noted that the fifth mechanism (Motivation to engage in social life and ability to project oneself into the future) emerged from the analysis but could not be explored in sufficient depth to identify its activation conditions. This is primarily because the mechanism involves desires and future projections. Because of their young age (8–15 years old) and disability, many CYWD struggled with abstract projection and were often not yet envisioning vocational training or adult life.

##### Three conditions for activating M1: Understanding the school environment.

###### Condition 1: Support is structured (C1_M1_):

The ingredients required to meet this condition are listed in Table 1.

**Table 1:** Combination of ingredients required to meet Condition 1 (C1_M1_).

Ingredients	Influenced by
Personalized weekly schedule that integrates the different times and places of schooling (CiDAME1)	• Effective coordination between the National Education system and DAME (CoDE3) • Sector coordinator (CiDAME46) • Initial training (CeDAME5) and prior experience of DAME professionals (CeDAME6) when these promote skills in formalization and written communication
Tools to facilitate the visualization, understanding, and memorization of the schedule (CiDAME3), used by all professionals working with the CYWD	**•** Effective coordination between National Education and DAME
Specific rooms assigned to each activity (CiDAME8) to help the student adopt the appropriate posture according to the context	• Strengthened partnerships with local authorities (CiDL19) that provide facilities or spaces (CiDL21)
Routines consistently followed (CiDAME4)	**•** Presence of a sector coordinator responsible for planning all DAME interventions (CiDAME46) **•** Flexibility in DAME organization: any professional can substitute for another (CiDAME47) **•** In case of replacement, continuity of support is ensured: long-term support of CYWD by DAME (from ages 6–15 years) (CeDAME1); all DAME professionals know every CYWD

###### Condition 2: Support provided by the various stakeholders is coherent, and this coherence is made explicit to the CYWD (C2_M1_):

The ingredients required to meet this condition are:

- A shared educational and pedagogical project that underpins professional practices and supports their joint evolution (CoDE1); DAME professionals, in addition to supporting CYWD, also play a support/resource role for teachers (CiDAME9–35)
- Multidisciplinary teamwork between institutions (CoDE3)
- Shared tools (e.g., a crisis management protocol for CYWD) (CoDE2)

This continuity of activities across different professionals is sometimes hindered by unfilled positions within DAME (CeDAME3)—particularly therapist positions—as well as by the precarious working conditions of AESH, staffing shortages, and resulting turnover (CeEN14). Continuity should ideally extend to other professionals as well (e.g., therapists outside DAME) and, in some cases, to professionals supporting the CYWD within other medico-social structures (e.g., specialized disability services) or other sectors (e.g., Child Welfare Services). It may also be threatened by the instability of these professionals (CeDAME8).

###### Condition 3: The school pathways of CYWD prioritize continuity and anticipate transition situations (C3_M1_):

The ingredients required to meet this condition are listed in Table 2.

**Table 2:** Combination of ingredients required to meet Condition 3 (C3_M1_).

Ingredients	Influenced by
Schooling in a mainstream environment from an early age, starting in preschool (CiED2)	**•** Principle of compulsory schooling in the local catchment school (CiED1)
Follow-up with the reference class (CiED11): the CYWD therefore keeps the same classmates whom he/she knows and who know him/her	**•** Principle of schooling in the age-appropriate class or N + 1 (CiED3)
Continuity of support by the same DAME during transitions (e.g., from primary to secondary school), or—if a change is planned due to age groups—anticipation is possible because contacts can be established beforehand between structures (CiED12)	• Follow-up of CYWD by DAME according to age groups (e.g., 6–15 years) (CeDAME1) • Sector-based organization of DAME (CiED16)

##### Four conditions for activating M2: Feeling like a student.

###### Condition 1: Feeling like a student belonging to the school (C1_M2_):

The ingredients required to meet this condition are listed in Table 3.

**Table 3:** Combination of ingredients required to meet Condition 1 (C1_M2_).

Ingredients	Influenced by
Schooling in the local catchment school (CiED1) and support by DAME within that school (CiED4)	
School time of the CYWD in the reference class prioritized as much as possible over DAME support time outside the classroom (CiED7)	
Participation in school trips, outings, and events, as adaptations are planned and anticipated (CiEN3)	
Option to have lunch in the school cafeteria, if parents wish, in the same dining room as other students (CiED14)	Management staff made aware of and trained in disability (CiED20), supported by partnerships with local authorities (CiED19)
Access to the leisure center without additional administrative procedures (CiED15)	Disability Resource Team at the departmental level (CiED22)

###### Condition 2: Feeling like a student of the teacher (C2_M2_):

The ingredients required to meet this condition are listed in Table 4.

**Table 4:** Combination of ingredients required to meet Condition 2 (C2_M2_).

Ingredients	Influenced by
Sufficient time spent in class, as schooling time is prioritized (CiED7)	
Proper functioning of the class in the presence of the CYWD (CiEN10)	• No disruptions linked to the presence of the CYWD (CiEN8): 1. Appropriate and adapted schooling setup for the CYWD to prevent inappropriate behaviors stemming from discomfort (CiEN1); 2. Support by DAME in class according to the CYWD’s needs (CiDAME5), reinforced by the availability and stability of DAME support resources (CiDAME45) and AESH (CeEN14); 3. Discretion of professionals working in class with the CYWD, to avoid distracting the class (e.g., oral instructions) (CiDAME36); and 4. Skills of the teacher and accompanying professionals in managing challenging behaviors (CoDE5) • Benefit for the class (CiEN9): 1. Support from DAME professionals and AESH for other students in need (CiEN11); 2. Pedagogical adaptations planned for the CYWD also offered to other students (CiEN12); and 3. Co-teaching strategies between the classroom teacher and the specialized teacher (CiEN13) • Class size and composition (CeEN1), which can be challenging when many children have varied difficulties
Time dedicated by the teacher to the CYWD to get to know them and monitor their progress (CiEN16)	• Co-teaching strategy (CiEN13): the DAME teacher leads the class while the classroom teacher teaches the CYWD
Main contacts for the CYWD and their family: teachers and the school (CiEN4)	• Families guided by DAME professionals to the teacher for direct contact (CiDAME40) • Difficult situations or conflicts managed directly with the school (CiEN5), with DAME intervening only to provide a contributive perspective • Parents encouraged by DAME professionals to attend parent–teacher meetings at the school or secondary school (CiDAME41) • CYWD dropped off in the morning by their parents (CiEN2), made possible by schooling in the local catchment school (CiED1)

###### Condition 3: Feeling like a member of the class (C3_M2_):

The ingredients required to meet this condition are listed in Table 5.

**Table 5:** Combination of ingredients required to meet Condition 3 (C3_M2_).

Ingredients	Influenced by
Support from DAME professionals and AESH for other students in need (CiEN11) Pedagogical adaptations planned for the CYWD also offered to other students in the class (CiEN12) Educational sessions initially planned for the CYWD outside the classroom also offered to other students in need (CiDAME37)	• Good team coordination (CoDE3) • Availability of rooms within or near the school (CiED21), supported through partnerships with local authorities (CiED19) • Individual nature of human-assistance compensations notified by the county-level public agency (“*MDA”*) (CeDAME2): the professional must focus exclusively on the CYWD. This obstacle is overcome through the ability of support professionals to deviate from standard rules (CiDAME57)
Sufficient time spent in class, as the CYWD’s time in their reference class is prioritized (CiED7)	
Autonomy in class and at school encouraged by limiting DAME or AESH support as much as possible (CiED8)	
CYWD works from the same materials as classmates, with objectives adapted to their needs (CiEN17)	
CYWD seated next to another student (CiEN7), with the seat not reserved for the AESH	
Opportunity to participate in all class activities without logistical barriers (CiEN3)	• Teamwork between National Education professionals and DAME professionals (CoDE3)
Same “attributes” as other students (CiEN7): name on the class list, school communication notebook, inclusion in the class photograph	

###### Condition 4: Feeling like a classmate (C4_M2_):

The ingredients required to meet this condition are listed in Table 6.

**Table 6:** Combination of ingredients required to meet Condition 4 (C4_M2_).

Ingredients	Influenced by
Follow-up with the reference class (with the same classmates) (CiED11)	**•** Principle of schooling in the age-appropriate class (CiED3)
Opportunities for interactions between classmates encouraged and maintained (CiEN14)	**•** No seat reserved for the AESH next to the CYWD (CiEN6) **•** Possibility to take part in all class activities, as accommodations are planned in advance (CiEN3)
Support from the DAME to help the CYWD develop a classmate posture (CiDAME5)	

##### Three conditions of activation of M3: Acknowledging one’s own difference.

###### Condition 1: A tailored schedule and individualized support (C1_M3_):

The ingredients required to meet this condition are listed in Table 7.

**Table 7:** Combination of ingredients required to meet Condition 1 (C1_M3_).

Ingredients	Influenced by
Balance sought between time spent in class and outside class (CiED7), between autonomy and support (CiED8), and consideration of the CYWD’s high fatigability (CiED9)	• Time for release/break/nap integrated into the schedule (CiDAME2), requiring an appropriate school layout (CiED21)
Minimization of the CYWD’s transport time (CiED10)	**•** DAME support (therapeutic, educational) provided close to the living environment (CiED6)
Focus on processes of students’ needs and expectations (CiED13)	• Tailored and holistic schedule (CiDAME1) established through priorities: 1. Multidisciplinary teamwork between National Education and DAME (CoDE3); 2. Sector coordinator (CiDAME46); and 3. Involvement of the DAME service manager (CiDAME54) • In-depth knowledge of the CYWD by the DAME, which supports the child over several years (DAMEs are organized by age groups: 0–6 years, 6–15 years, ≥ 16 years) (CeDAME1), while other professionals may work with them for shorter periods • Parents involved in this planning process (CiDAME43)

###### Condition 2: Flexibility in the schedule and DAME support to adapt to the CYWD’s development and daily, weekly, or life-period experiences (CiDAME6) (C2_M3_):

The ingredients required to meet this condition are:

- Coordination between National Education and DAME teams (CoDE3)
- Shared tools to streamline information flow and decision-making on scheduling (CoDE2)
- Organizational flexibility within the DAME (CiDAME47)

It is noted that the structuring of school time by academic year and periods (CiEN2) may conflict with the longer-term developmental timeline of the CYWD.

###### Condition 3: Adjustments designed to remain adaptable to each individual case (C3_M3_):

The ingredients required to meet this condition are listed in Table 8.

**Table 8:** Combination of ingredients required to meet Condition 3 (C3_M3_).

Ingredients	Influenced by
Adapted rooms for DAME activities (educational or rest) (CiED21)	• Partnerships established with local authorities
Equipment adapted to the CYWD for educational activities provided by the DAME (CiDAME7)	
DAME staff available for tailored support in mainstream settings (CiDAME45)	

##### Five conditions for activating M4: Motivation to engage in learning.

###### Condition 1: Feeling like a student of the teacher (C1_M4_):

This condition corresponds to Condition 3 of Mechanism 2 and has been described above.

###### Condition 2: Feeling recognized as a student capable of learning differently (C2_M4_):

The ingredients required to meet this condition are listed in Table 9.

**Table 9:** Combination of ingredients required to meet Condition 2 (C2_M4_).

Ingredients	Influenced by
Recognition that, in terms of learning (content, pace, level, progression), the path of a CYWD may differ greatly from that of other students (CiEN18)	
Adjustment of expectations to meet the student where they are and build on their abilities. Establishment of long-term, prioritized, and adapted goals, detached from the standard curriculum objectives for students of the same age (CiEN19)	• Possibility of implementing a “*programmation adaptée des objectifs d’apprentissage”* (PAOA) (adapted learning objectives plan) (CeEN6)
Creation of solutions for each situation through a trial-and-adjustment approach, with regular updates to materials and pedagogy (CiEN20)	
Use of UDL principles (CiEN25)	• Teacher training in UDL (CiEN29)
Creation of conditions that enable the student to make progress (CiEN22): balancing risk and protection	

###### Condition 3: Feeling valued (C3_M4_):

The ingredients required to meet this condition are:

- Emphasis on the CYWD’s abilities (CiEN23)
- Identification and recognition of even minimal progress by the CYWD (CiEN21), with public acknowledgment in class
- Creation of solutions for each situation through a trial-and-adjustment approach, with regular updates to materials and pedagogy (CeEN20)
- Assignment of valued roles to the CYWD by the teacher (CiEN24), giving them tasks—completed independently or with help—just like other students

###### Condition 4: Feeling recognized as a student with potential (C4_M4_):

The ingredient required to meet this condition is a positive attitude on the part of the teacher toward the CYWD (CiEN29). The teacher believes in the student, allows their potential to emerge, and does not limit opportunities for progress through preconceived ideas.

###### Condition 5: Finding meaning in school and learning (C5_M4_):

This condition is present as soon as Mechanism 2 *Feeling like a student* is activated. The CYWD engages in shared group activities, experiences emotions and connections with peers, and becomes part of a collective learning dynamic. These experiences help them build understanding and reference points, supporting the development of meaning in school and in learning.

### Study conclusions

Based on the results presented in this study, the following conclusions can be directly supported by the empirical evidence.

#### How does inclusive education work and under which conditions?

##### Inclusive education (learning, autonomy, participation) relies on five interrelated mechanisms in CYWD.

The research shows that five mechanisms must be activated in CYWD to promote learning (O1), autonomy (O2), and social participation (O3). These mechanisms are the ones that must be reproduced in every context. Drawing on J. Bowlby’s attachment theory [38,39], we propose an articulation of the five mechanisms identified in the study. The first three mechanisms (M1 *Understanding the school environment*, M2 *Feeling like a student*, and M3 *Acknowledging one’s own disability*) interlock chronologically with the fourth and fifth mechanisms (M4 *Motivation to engage in learning* and M5 *Motivation to engage in social life and ability to project oneself into the future*). CYWD must first mobilize cognitive resources to become familiar with their environment and to feel safe, experiencing themselves both as students and as persons with a disability. Only then can they release resources for exploration mechanisms—engagement in learning and participation or projection into social life, both professional and civic. As Bowlby [38,39] noted, a *secure base enables exploration* because the assurance of safety frees cognitive and emotional resources for curiosity and social engagement.

It is also worth noting that mechanisms M2 *Feeling like a student* and M3 *Acknowledging one’s own difference* exist in a delicate balance. This involves maintaining two identities and shifting between them over time—learning to navigate multiple identities. There is a dynamic interplay between belonging and respect for difference. Others should treat CYWD respectfully as ordinary students, while also recognizing their challenges and providing support discreetly, thus preventing them from being overwhelmed by the demands of a new, multifaceted environment without drawing unnecessary attention to the challenge. This connects to broader discussions about whether it is the individual who adapts to the environment or the environment that adapts to the individual—an ongoing balance. It echoes Fougeyrollas’s work on a psychoeducational approach, which emphasizes the importance of balancing personal adaptation (with the risk of conformity) and environmental adaptation [40].

##### Conditions required to activate the five mechanisms: insights into the complex dynamics of inclusive education:

Realist evaluation makes it possible to identify with precision the conditions required to activate mechanisms. The research shows that 15 success conditions—composed of 133 contributing ingredients—must be gathered. The next chapter highlights some of the key ingredients emerging from the results.

**Fundamental principles of inclusive education.** The study identified 15 ingredients related to the schooling modalities established in the Eure-et-Loir model. These ingredients appear across four mechanisms and eight conditions (C3_M1_, C1_M2_, C2_M2_, C3_M2_, C4_M2_, C1_M3_, C1_M4_, and C4_M2_), reflecting their centrality to the achievement of inclusive education. They can be grouped into three fundamental principles:

- CYWD should attend school to the maximum extent appropriate and be the student of the regular teacher (CiED1–3, 5, 7, 8, 11)
- CYWD should receive the tailored specialized support they need, primarily provided within their everyday life environment. (CiED4, 6, 9, 10, 12, 13)
- The initiative should address not only the education system but also spaces and times beyond it (lunch time, afterschool programs, extracurricular activities, and more) (CiED14, 15).

These principles guide many of the choices and conditions that shape inclusive education.

###### Cooperation between disability-specific and mainstream education professionals:

The results show that cooperation between specialized-sector professionals and those from the national education system is central to daily practice. This cooperation operates at several levels:

i Classroom functioning: CYWD often require human support (teaching assistants, educators, specialized teachers). For this support to be effective, professionals must be able to work collaboratively within the same classroom.
ii Teacher learning and professional development: specialized professionals transfer knowledge and expertise from special education to mainstream teachers (CiDAME27–32).

Strong partnerships must therefore be forged between service providers and the national education system. A first requirement is the development of a shared understanding of inclusive education grounded in the conviction that schooling has a dual objective: academic achievement and the development of psychosocial skills. This shared vision supports an integrated approach and collaboration toward a common agenda. Several practices emerge as key, such as co-teaching (CiEN13), UDL (CiEN52), the use of communication tools (CiDAME3), cross-training between National Education and specialized services (CiEN44), and inter-institutional meetings (CiCoDE3). This cooperation calls for a rethinking of professional identities and roles.

The study also highlights that such cooperation is not easy to implement—lack of time (CeEN5) and lack of dedicated spaces often make collaboration difficult. However, some responses are emerging, such as dedicating part of teachers’ council meetings to inclusive education (CiEN33).

**A systemic approach.** Cooperation between National Education professionals and specialized professionals must be supported by an appropriate framework and a favorable environment at all levels. This is a systemic issue. This is demonstrated by the number of ingredients linked to organizational or structural dimensions (e.g., within the National Education category, 42 ingredients [CiEN27–52, CeEN1–16] out of 68). No single approach is sufficient to drive change; rather, multiple interconnected factors are critical to the success of inclusion efforts.

**A territorial approach.** The findings show that many conditions depend on factors rooted in district-level decisions and on bringing local mainstream stakeholders (e.g., local authorities, sports associations) into the partnership. The 22 ingredients pertaining to the Eure-et-Loir district model (CiED1–22) appear 33 times across the conditions. This means these ingredients have an average use of 1.5 times, whereas the ratio is equal to or below 1 in other ingredient categories. For example, the ingredient “Partnership with municipalities” (CiED19) appears in three mechanisms (M1–M3) and four conditions (C1_M1,_ C2_M2,_ C3_M2,_ C3_M3_).

#### The powerful potential of the conditions of activation for action.

**Fig 1:**
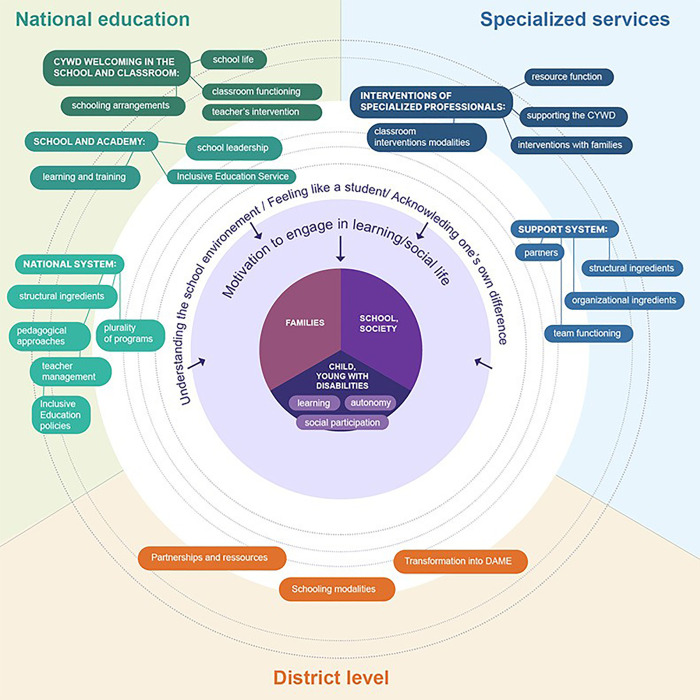
Inclusive school conceptual framework.

The results enabled us to develop a conceptual framework that highlights the mechanisms to activate and the success conditions to gather (Fig 1). The framework shows that inclusive schooling can be effective when supported by substantial resources, structured approaches, and strong partnerships. However, such conditions are generally not met in practice today. It is therefore essential to give due consideration to the conditions that must be brought together to make inclusion effective. Depending on their specific context, stakeholders can assess which conditions are already in place and which require strengthening. The results make it possible to determine the combinations of elements needed to create these conditions, mobilize identified levers, and enrich reflection with insights drawn from their own context. Notably, this active reflective process fosters collective thinking, stakeholder buy-in, and the anchoring of actions in the realities of the field.

It is noteworthy that the same ingredients can, depending on the circumstances, either support or hinder certain conditions and mechanisms. For example, support provided by specialized professionals is required for the three conditions needed to activate Mechanism M3 *Recognizing one’s disability*. Conversely, Condition C3_M2_ *Being the student of the teacher*, which includes reducing this support (CiDAME8), is necessary to activate Mechanism M2 *Feeling like a student*. Such tensions have already been reported in the literature [41]. In this sense, a professional may unintentionally act to the detriment of a mechanism. This underscores the need for professionals to act knowingly, with the explicit intention of activating a mechanism. From this perspective, the findings generated by realist evaluation are particularly powerful because they specify for each ingredient the key mechanisms to which it is linked and provide concrete understanding and guidance for taking action without losing sight of the intended purpose.

It should also be emphasized that the policy environment is evolving very rapidly. New laws and frameworks are regularly introduced by the Ministry of National Education and must be integrated and articulated by local stakeholders within their existing modes of functioning. A recent example in France is the introduction, at the start of the 2025 academic year, of the *Pôle d’Appui à la Scolarité*, intended to offer prompt and adapted first-line responses to children with specific needs. The strength of realist evaluation lies in its ability to account for such contextual changes and to allow CMOs to evolve in real time alongside developments in the field.

## Interpretation and implications

Beyond the empirical findings, the following section discusses possible interpretations of the results and their broader implications.

### Is inclusive education beneficial?

#### Opening possibilities and challenging preconceived limits.

The findings of this study indicate that families and professionals often observe unexpected gains among children with disabilities (O1). Similar outcomes have been documented in previous research [16]. It is well recognized that children with disabilities are at significant risk for limited participation across multiple settings compared with their typically developing peers—including preschool and school (e.g., [42]), home (e.g., [43]), and afterschool activities (e.g., [44]). Meanwhile, the frequency and quality of social relationships are key determinants of a child’s development [45]. Children should therefore be encouraged to develop and practice social skills through engagement in diverse social contexts—such as schools, community associations, and leisure activities of their choosing [46]. Participation in varied contexts fosters social learning through observation of peers, social comparison, and active interaction [47]. These environments provide opportunities to practice social skills that may be less challenged within the highly structured routines of specialized settings and may remain underdeveloped when participation is restricted. Developing such skills early is crucial because performance expectations and developmental gaps widen with age, increasing the risk of exclusion from activities [48]. Participation in community settings beyond school also reduces the emphasis on performance and competition, allowing for greater diversity in abilities and modes of engagement. Interacting with different adults and peers, and in environments with fewer formal rules, broadens children’s understanding of everyday contexts, strengthens adaptability, and encourages exploration of new activities and experiences [48]. This supports, as illustrated in the French district of Eure-et-Loir, the importance of promoting inclusion as early as possible—both in school and beyond—as it helps prepare CYWD for adulthood by providing opportunities to explore new activities and interactions and to practice the skills needed in mainstream environments.

One expected long-term effect of inclusive education is the expansion of CYWD’s capacity to envisage professional futures without preconceived limitations. CYWD are rarely exposed to working adults with disabilities. In France, for instance, teachers with disabilities are significantly underrepresented [49]. As a result, CYWD seldom encounter disabled adults in professional roles in their day-to-day schooling, which limits their opportunities to imagine themselves in such positions. Yet inclusive education creates conditions for training a growing number of CYWD [50] for a wide range of careers. Over time, more CYWD may enter the teaching profession, providing future generations with meaningful role models.

Inclusive schooling also influences changes in family social integration (O5). Families must navigate the stigma associated with their child’s disability [51], and they are sometimes marginalized by their social environment. When their child is enrolled in mainstream education, parents become “parents of a student” rather than parents of a child educated in a specialized setting. They are no longer stigmatized by the place of their child’s schooling. For example, they may now take their child to the local school (CiEN2) rather than relying on a taxi service, creating opportunities to build relationships with other parents and to meet teachers. In addition, in families of children with disabilities, daily life often centers on medical appointments and interventions, which can lead to viewing the child primarily through the lens of disability and support needs [52]. Becoming a student allows parents to see their child not only as disabled but also as a person with many dimensions. Parental perceptions can shift—they may see their child as capable, believe in their potential, and envision their future. Such changes can have a substantial impact on CYWD.

Choosing a schooling arrangement for their child (O4) is not straightforward for families, and inclusive education still generates concerns, particularly for children with neurodevelopmental disorders [2]. We can assume that the more families are able to exercise their rights, the more empowered they will become in this process and, through a snowball effect, the more likely they will be to encourage other families to do the same.

#### Shaping the society of tomorrow.

This study supports previous findings [53] showing that the inclusion and schooling of students with disabilities help foster a positive view of differences among all students (O6). Over time, it is likely that these students will become adults with more favorable and positive attitudes toward disability, which could, in turn, reduce stigma. The literature has shown that one factor influencing attitudes and perceptions toward disability is the amount of prior contact individuals have had with persons with disabilities [45,54,55]. In France, the statistical likelihood of encountering a student with a disability in a mainstream classroom has been steadily increasing: the number of students with disabilities enrolled in regular education tripled between 2006 and 2022 [56], meaning that increasingly more people will be familiar with disability. It can therefore be reasonably assumed that education for all will have a profound long-term impact on society because children with disabilities will no longer be segregated into specialized settings. Young people will now experience schooling alongside CYWD. Informal peer interactions in inclusive schools are powerful spaces for learning about disability and create opportunities to address what Régine Scelles calls the “fundamental enigma of disability” [57]. They allow children to build equal relationships, explore difficult questions, and make difference familiar. While informal interactions are key, the present results show that specific intervention targeting this group could synergize with this process. Indeed, classmates reported a need for dedicated spaces and a designated interlocutor to discuss matters related to disability—findings consistent with previous research [33]. Overall, we argue that inclusive systems play a crucial role in transforming discriminatory attitudes.

#### When inclusion is not going well: first check whether conditions for success are in place.

Inclusive education is often measured by the number of CYWD enrolled in school. The present research helps document and specify the real effects in CYWD that should be expected and assessed beyond mere physical presence. For some CYWD, inclusion has clear positive effects; for others, the benefits are less visible and require careful observation of subtle signs. Inclusion is a dynamic, time-based process that varies for each child. The results show that unexpected gains may arise, with no predictable pattern—supporting the principle that all CYWD should be given the chance and the time to benefit from inclusive education (consistent with the rights-based approach promoted by the UNCRPD). At the same time, professionals and families must remain vigilant and responsive to individual situations. Beyond individual effects, inclusion produces outcomes for families, peers, and society at large. It helps challenge stereotypes and prevents the unintended narrowing of children’s horizons. By fostering enabling environments and shifting representations of disability, inclusion creates a virtuous circle. This study underlines that inclusion is not a question of *if* but of *how*. It requires sustained effort, careful monitoring, and concrete support of various kinds.

### Limitations

This research acknowledges several limitations. The effects on CYWD are difficult to measure because they are subjective and vary from child to child. Gathering the perspectives of CYWD is also challenging (see Methods section). Moreover, we were unable to identify the conditions required to activate Mechanism 5.

It would be valuable to explore further the role of CYWD and their families in schooling—for example, by more closely examining their involvement in the development of Personalized Schooling Projects (*Projet Personnalisé de Scolarisation*), their participation in Schooling Support Team (*équipe de suivi de scolarisation*) meetings, their relationships with specialized teams, their attendance at parent–teacher conferences, and their interactions with the school team. Some relevant ingredients emerged in the study but were not presented here.

It is important to recognize that this study provides only a partial understanding of how inclusive education works and how its many components interact. The research was conducted in two cases—one primary school and one middle school. It is likely that numerous contextual factors influence inclusive schooling and its effects. Expanding the study to additional cases would help clarify how different contexts shape implementation (e.g., urban vs. rural areas, public vs. private education). Examining preschool and high school settings would also shed light on the full educational trajectory of CYWD, as transition periods are critical. Consistent with the cyclical nature of a realist approach, the CMOs identified here must be confirmed or refined through future empirical studies.

## General conclusion

Realist evaluation offers a comprehensive approach for assessing interventions in complex situations with a view toward transferability. We conducted a study that unpacked the inclusive education initiative developed through a partnership of key stakeholders in France. This study shows that it is possible to implement high-commitment inclusive school practices at a district level and that these practices are perceived to have an effect on CYWD. The results provide valuable insights and operational considerations for policymakers, educators, and researchers, paving the way for innovative approaches and strengthened inclusive educational practices.

The findings contribute to ongoing debates about inclusive schooling. Indeed, there is a risk of concluding that inclusion is not beneficial when the necessary conditions are not met. By providing precise insights into the conditions that must be in place, this study helps avoid premature conclusions based on non-relevant criteria. On the contrary, it supports the development of increasingly refined, relevant, and comparable measures, along with practical tools to guide action.

The findings contribute to ongoing debates about inclusive schooling by clarifying the configurations required for inclusion to function as intended. They suggest that when key conditions are not assembled, inclusion may fail to generate the expected experiences and benefits. By specifying these conditions, the study supports more precise evaluation criteria and provides operational guidance for policymakers, educators, and researchers seeking to strengthen inclusive educational practices..

## Supporting information

S1 Table Translation of UNCRPD principles into potential mechanisms. (DOCX)

S2 Table CYWD interviews adaptations. (DOCX)

S3 Table Breakdown of interview participants in each setting. (DOCX)

S1 File Rameses II cheklist. (DOCX)

S2 File Interview guides. (PDF)

S3 File Interventional and contextual ingredients. (PDF)
