# Supplementary material for: Understanding the conditions for inclusive education: A realist evaluation of a French territorial innovation
Source: PLoS One. 2026 Apr 29;21(4):e0348203. doi: 10.1371/journal.pone.0348203 (PMC13128107; doi:10.1371/journal.pone.0348203)
Supplement: S1 Table — (DOCX) [file pone.0348203.s001.docx]

**S1 Table. Translation of UNCRPD principles into potential mechanisms.**

| Principles of the Convention | Expected Mechanisms |
| --- | --- |
| • Respect for inherent dignity, individual autonomy including the freedom to make one’s own choices, and independence of persons  • Non discrimination  • Full and effective participation and inclusion in society  • Respect for difference and acceptance of persons with disabilities as part of human diversity and humanity  • Equality of opportunity  • Accessibility  • Equality between men and women  • Respect for the evolving capacities of children with disabilities and respect for the right of children with disabilities to preserve their identities | • Feeling in control (as opposed to loss of control)  • Perceiving oneself as not being hindered  • Feeling self-efficacious  • Feeling self-confident  • Feeling treated with dignity  • Feeling valued  • Feeling recognised (as a person) / feeling considered  • Feeling useful, contributing to society  • Feeling a sense of belonging to the community  • Feeling respected |
