# Supplementary material for: Understanding the conditions for inclusive education: A realist evaluation of a French territorial innovation
Source: PLoS One. 2026 Apr 29;21(4):e0348203. doi: 10.1371/journal.pone.0348203 (PMC13128107; doi:10.1371/journal.pone.0348203)
Supplement: S2 Table — (DOCX) [file pone.0348203.s002.docx]

**S2 Table. CYWD interviews adaptations**

| Phase | Adaptations/challenges |
| --- | --- |
| Preparation | - Development of interview guides: The guides were co-designed with specialized teams to ensure that questions were phrased in clear, simple language. - Bias mitigation: To reduce bias related to partial inclusion or social isolation (e.g., students with disabilities are rarely chosen as friends by their peers [1,2]), questions were carefully worded. For example, instead of asking, “Do you have friends in the playground?” the question was phrased as, “Who are your friends in the playground?” - Adaptations during interviews: Several tools were used to support communication and engagement, including visual aids (class photos, schedules, pictograms), emotion-expression tools (emotion cards, smiley scales), and focus aids (fidget toys, timers). - Environment: Attention was given to choosing a familiar, low-stimulation room. - Support and trust: Interviews were conducted alongside a DAME professional to help create a trusting atmosphere and a psychologically safe space for open discussion. |
| Information transfer | - Some students displayed social desirability bias and sought to please the adult interviewer. - Others provided limited verbal elaboration, had difficulties with temporal orientation, or struggled with abstract projection. Only questions directly linked to recent lived experience were asked; questions requiring projection were omitted when necessary. - Fatigue and attention: Many students tired quickly or had difficulty concentrating. Interviews were therefore adapted in duration and rhythm to suit each student. Fidget tools and timers were used to help structure the sessions. |
| Interpretation | - Interviews with students with disabilities were always followed by a debriefing session involving a psychologist and the student’s referring teacher, to collaboratively interpret the responses. Although some interviews could not be very deep or exploratory (due to the constraints described above), the specialized professionals’ in-depth knowledge of each child allowed for more contextualized and meaningful interpretation. - In addition, specialized staff who were well acquainted with the students’ backgrounds provided valuable insights into biographical trajectories (e.g., family events, schooling history, disability narratives) and life contexts (e.g., household composition, residential status, such as group homes). - In certain cases, these elements were cross-validated through (i) interviews with the students’ parents, classmates, teachers, and support staff and (ii) informal conversations with pedagogical coordinators or other professionals during site visits. |
