# Supplementary material for: Understanding the conditions for inclusive education: A realist evaluation of a French territorial innovation
Source: PLoS One. 2026 Apr 29;21(4):e0348203. doi: 10.1371/journal.pone.0348203 (PMC13128107; doi:10.1371/journal.pone.0348203)
Supplement: S3 Table — (DOCX) [file pone.0348203.s003.docx]

**S3 Table. Breakdown of interview participants in each setting.**

**Breakdown of Interview Participants in the Primary School.**

| Interviewees | Number of Interviews | Number of Participants | Description |
| --- | --- | --- | --- |
| CYWD | 4 | 5 | One group interview (two students) and three individual interviews |
| Classmates from two reference classes | 2 | 10 | Group interviews with five students from each of the two reference classes |
| Families | 2 | 3 | Individual interviews |
| DAME professionals | 8 | 8 | Individual interviews: Head of Service, Pedagogical coordinator, Special Education Teacher, Psychologist, Sector coordinator, Specialized Educator, Sport Educator, Psychomotrician. |
| National Education professionals | 7 | 7 | Individual interviews: Deputy Principal, Teacher of an inclusive education support unit inside the school, three reference class Teachers, two support assistants for students with disabilities (“AESH”) |
| School life professionals | 2 | 2 | Leisure Center Coordinator, school life staff Member |
| Total | 25 | 35 | — |

**Breakdown of Interview Participants in the Secondary School.**

| Interviewees | Number of Interviews | Number of Participants | Description |
| --- | --- | --- | --- |
| CYWD | 3 | 5 | One group interview (three students) and two individual interviews (five students in total) |
| Classmates from two reference classes | 2 | 6 | Group interviews with three students from each of the two reference classes |
| Families | 2 | 2 | Individual interviews |
| DAME professionals | 5 | 5 | Individual interviews: Head of Service, Special Education Teacher, Educator, Driver, Educational Coordinator |
| National Education professionals | 5 | 5 | Individual interviews: Deputy Principal, teacher of an inclusive education support unit inside the school, two reference class teachers, two reference class teachers, one support assistant for students with disabilities (“AESH”) |
| School life professionals | 2 | 2 | Two school life staff members |
| Total | 19 | 25 | — |
