# Supplementary material for: Understanding the conditions for inclusive education: A realist evaluation of a French territorial innovation
Source: PLoS One. 2026 Apr 29;21(4):e0348203. doi: 10.1371/journal.pone.0348203 (PMC13128107; doi:10.1371/journal.pone.0348203)
Supplement: S2 File — (PDF) [file pone.0348203.s005.pdf]

S2 File – Interview guides

Interview guide dedicated to professionals

Mutual introductions<br><br>Reminder of the framework and objectives of the study.<br><br>The following themes are addressed:<br><br>• Understanding “school for all” and its effects on pupils and families: objectives and definition of inclusive education; link with the school project; role of the school versus specialized services; clarity of the inclusive school (role of each actor); benefits for pupils with disabilities; benefits for the whole school.<br>• Changes in professional practices (National Education and medico-social services): evolution of perceptions and professional identities; practices (skills, responsibilities); training; appropriation of tools and systems provided by specialized services; accessibility of teaching (attitudes, tools, adapted materials); accessibility of physical spaces and other adjustments; systems and tools supporting transformation and opening toward mainstream settings; provision of resources for National Education (awareness-raising, tools, guides, support).<br>• Implementation of the partnership between National Education and medico-social services: local networks; clarity of roles; reference persons; mutual knowledge and proximity between actors; transfer of know-how; ease of network mobilization; effectiveness of cooperation; existence and stability of established mechanisms; resource support; limits; expectations.<br>• Territorial dynamics: supporting factors from local dynamics (as triggers, in terms of pace, acceptability, or stability of transformation); obstacles and barriers.

Interview guides dedicated to families

General objective of data collection from families

The aim is:

- to identify what changes the “school for all” system has brought to pupils and their families—what it has provided, modified, or strengthened;
- to investigate what factors have enabled these changes in pupils and families.

**Mutual introductions**<br><br>**Reminder of the framework and objectives of the study.**<br><br>**Questions**

What is your child’s school path?

- How is your child's schooling going, both in terms of learning and inclusion?
- Are you involved in decisions concerning your child's schooling? Which decisions, and in what way? Who are your interlocutors? Is it easy or difficult to discuss with them?
- What do you think of your child's timetable, between inclusive time and time supported by the DAME? Does it seem appropriate? Do you think the proposed support is adequate?
- Does your child have relationships or activities outside school (with family, neighbors, friends, through extracurricular activities, etc.)?
- How do you see your child's future? What school path do you imagine?
- Is it important for you that your child is enrolled in the local school? Why? What does it bring? Do you have concerns?

Interview guides dedicated to children/young people

Caution: The attached document presents the themes that researchers will explore. It is specified that these questions are not intended to be asked directly to pupils as written. They will be reformulated according to the child's age and, for pupils with disabilities, according to their specific needs, to make them simple and non-stigmatizing. The research will be conducted in collaboration with DAME professionals, especially their resource centers, to achieve this adaptation. The Easy-to-Read and Understand method and visual supports will be used, along with any other accessibility tools required for each child. It is noted that the support provided by the DAME includes social support for the child in school life, leisure activities, and beyond—these are familiar topics for them.

General objective of data collection from children with disabilities

The aim is:

- to identify what changes the "school for all" system has brought to children—what it has provided, modified, or reinforced;
- to investigate what has enabled these changes in each child or young person.

In the school setting:

- Ability to find the resource they need.
- School trajectory and transitions (between levels, from school to secondary, to vocational training; continuity or rupture).
- Personal project: are expectations expressed, heard, realistic?
- Level of involvement in decision-making.
- Actual opportunities available.

In class:

- Interest in learning.
- Ability to ask for help.
- Ability to face difficulties.
- Learning progress (with adaptations when needed).
- Oral participation.
- School progress and results (promotion, meeting cycle criteria, exams, access to training, continuation of schooling in adolescence).
- Sense of belonging to the class (e.g. being chosen for a sports team, sitting next to a friend on a school trip).

Break times and lunch:

- Sense of belonging to the school (playing in the yard with peers, eating with friends in the cafeteria).

Outside school:

- Being invited to a classmate’s birthday or snack (participating in social rituals).
- Being invited by neighborhood or club friends.
- Participation in sports or cultural associations.
- Social appropriateness of behaviors.

In terms of projection and identity:

- Imagining one’s school journey.
- Imagining a professional project (for secondary students).
- Imagining adult life.
- Perceiving oneself as a pupil/citizen/student or as “disabled.”

For children/young with disabilities

Mutual introductions

Reminder of the framework and objectives of the study.

Questions :

- How is school going for you? Do you like going to school? Do you feel comfortable in class?
- Is learning easy or difficult?
- How is it going with your teacher? Do you do the same work as the others? How would you like it to go?
- Do you have any adjustments to make school easier? Do you have an AESH (support assistant)?
- During which times are you with your classmates? Would you like to be in class more or less often?
- If applicable: Do you prefer being in the specialized class?
- Do you think you need something else to help you?
- How do you feel with your classmates? Do you have many friends? Do you like recess and lunch times?
- In general, do you have any difficulties at school?
- What would you need to make it easier?
- Who do you go to if you have a problem at school?
- How do you see your schooling in the future? What do you want to do as an adult?
- Outside school, do you have extracurricular activities? How is that going?
- Outside school time, do you see friends? Are they school friends, neighbors, or friends from other activities??

For children/young without disabilities

Mutual introductions

Reminder of the framework and objectives of the study.

Questions

- Are you friends with a classmate who has a disability? Do you feel comfortable with them?
- Have you noticed any differences?
- Do you find it difficult to interact with them?
- If yes, why? In which situations is it complicated? What could help you?
- Do you think they experience difficulties?

- Do they fully belong to the class as pupils of the class?
- If not, what prevents that? What could help improve that?

Does having pupils with disabilities in your class change anything? Does it bring something positive? Does it make something harder?
