# Supplementary material for: Understanding the conditions for inclusive education: A realist evaluation of a French territorial innovation
Source: PLoS One. 2026 Apr 29;21(4):e0348203. doi: 10.1371/journal.pone.0348203 (PMC13128107; doi:10.1371/journal.pone.0348203)
Supplement: S3 File — (PDF) [file pone.0348203.s006.pdf]

22 Ingredients at the District Level (22 CiED)

The schooling arrangements that stem from the EpT28 scheme

CiED1	The CYWD is educated in their local school.
CiED2	The CYWD is educated in a mainstream setting from an early age (nursery school).
CiED3	The CYWD is educated in their age-group or N+1 class (depending on their academic level).
CiED4	The CYWD is supported by the DAME in their local school.
CiED5	At the beginning of the school year, the CYWD is educated in their reference class for 15 days, without DAME support (except by special authorization).
CiED6	DAME support (therapeutic, educational) close to the living environment is prioritized.
CiED7	The CYWD's schooling time in their reference class is prioritized (compared with time supported by the DAME outside the classroom).
CiED8	In class, the development of autonomy is encouraged by limiting DAME or AESH support.
CiED9	Consideration is given to the high fatigability of the CYWD.
CiED10	Minimization of the CYWD's transport time.
CiED11	The CYWD follows their reference class.
CiED12	The CYWD keeps the same DAME during transitions (e.g. from primary to secondary school), or if a change is required due to age-group boundaries, DAME sectorization facilitates collaboration as contacts can be established beforehand between structures.
CiED13	A focus of processes on students' needs and expectations.
CiED14	The CYWD can have lunch at the school canteen (if parents wish), in the same dining hall as other students.
CiED15	The CYWD has access to leisure centers without additional administrative procedures.

Transformation into DAME

CiED16	Sectorization of DAME intervention areas
CiED17	Each school in the department is covered by a DAME

Partenariats et Ressources

CiED18	An effective partnership with the MDA for CYWD notifications
CiED19	A partnership with local authorities
CiED20	Supervisory staff — at the level of local authorities (during breaks and meal times) — made aware/trained
CiED21	Provision of suitable rooms in mainstream settings and adapted school buildings
CiED22	Dedicated Disability Resource Team to organize the reception of CYWD in leisure centers

67 Ingredients related to DAME (59 CiDAME, 8 CeDAME)

Ingredients related to the intervention of DAME professionals

Ingredients linked to the support of the CYWD

CiDAME1	A personalized and holistic weekly schedule that takes into account the balance between classroom and out-of-class time, autonomy/support, and the high fatigability of the CYWD.
CiDAME2	Scheduled and clearly identified nap, rest, or relaxation times in the timetable.
CiDAME3	Tools to help the CYWD visualize, understand, and memorize the schedule (pictures, photos, pictograms, etc.) are created and used.
CiDAME4	The schedule is followed, meaning that routines are respected.
CiDAME5	The CYWD is supported by the DAME to develop psychosocial skills related to their role as a student and peer.
CiDAME6	Flexibility in the schedule and DAME support to adapt to the CYWD's development and experience.
CiDAME7	Available materials
CiDAME8	Adaptaed rooms

Ingrédients related to the resource function for teachers

	Reassuring teachers about the DAME's role.
CiDAME9	Be friendly, considerate, and proactive toward teachers by checking in regularly ('How are things going in your class?').
CiDAME10	Avoid being judgmental
CiDAME11	Be approachable to meet needs.
CiDAME12	Wait to be approached by the teacher at the beginning of the year; avoid suggesting demanding adaptations.
CiDAME13	Early in the year, DAME professionals may themselves create adaptations (especially specialized teachers).
CiDAME14	Gradually increase engagement according to the situation.
CiDAME15	Respond positively to teacher requests even when all seems fine.
CiDAME16	Highlight successful initiatives and clarify professional roles.
CiDAME17	Be clear about the skills of each professional (particularly pedagogical versus educational). Tension: the boundary between the mandates of each profession is sometimes blurred in real situation
CiDAME18	Be vigilant about your mission; blurred roles can cause inappropriate situations.
CiDAME19	Recognize that relationships are key.
CiDAME20	Be part of the school team (email, presence in staff room, meetings, etc.).
CiDAME21	Establish a link with school management and take part in school life.
CiDAME22	Get involved in the life of the school or secondary school (e.g. take part in events and informal moments).
	Show DAME credibility: efficiency and responsiveness.
CiDAME23	Help teachers identify their needs to adapt pedagogy.
CiDAME24	Provide small, concrete solutions.
CiDAME25	Respond quickly to any request.
CiDAME26	Act immediately in urgent situations.
	Transfer skills to teachers to empower them to adapt independently.
CiDAME27	Share precise information on the CYWD's situation and needs.

CiDAME28 Show possible adaptations.
CiDAME29 Reassure teachers when attempts don’t work.
CiDAME30 Promote creativity and testing rather than one-size-fits-all rules.
CiDAME31 Explain that adaptation is professional growth, not extra workload, and DAME remains supportive.
CiDAME32 Share principles of Universal Design for Learning (UDL).
Co-construction with the teachers (concerns specialized teachers)
CiDAME33 Co-design and co-teach with teachers.
CiDAME34 Take time to plan lessons.
CiDAME35 Support school leadership and staff.
* certains de ces items s'adressent plus particulièrement aux enseignants spécialisés
***En cas de forte résistance de l'enseignant, faire valoir la notion de droit ouvert (suite à la notification MDA) et a minima être présent. Exceptionnellement demander une dérogation pour que l'ESH soit scolarisé dans un autre établissement.*

<u>Ingrédients related to classroom intervention modalities</u>

CiDAME36 Professionals supporting the CYWD are discreet and avoid disturbing the class.
CiDAME37 When organizing sessions outside class, also include other students with needs.
CiDAME38 DAME professionals and AESH support others; adaptations for CYWD benefit the whole class.
CiDAME39 DAME teachers co-teach in class.

<u>Ingredients related to interventions with families</u>

CiDAME40 DAME professionals direct families to teachers for direct contact.
CiDAME41 Encourage parents to attend school meetings and reassure them about inclusion.
CiDAME42 Provide clear information about schooling, canteen, and leisure centers.
CiDAME43 Involve families in planning the schedule.

Ingredients related to the support system

<u>Structural factors</u>

CeDAME1 DAME supports the child over several years (organized by age group: 0–6, 6–15, 16+).
CiDAME44 Decrease in professional contact time due to lost time, increased individual sessions, and travel.
CeDAME2 MDA human assistance notifications are individual; the professional supports only the CYWD.

<u>Organizational factors</u>

CiDAME45 Sufficient human resources are needed for quality support across many time slots.
CiDAME46 Creation of sector coordinator position
CiDAME47 Flexible organization for substitutions.
CiDAME48 Structured and formalized DAME activities ensure continuity.
CiDAME49 Provide adapted rooms and materials
CeDAME3 Unfilled positions
CeDAME4 Compliance with regulations (e.g. GDPR).

<u>At the team level</u>

	At the management team level
CiDAME50	Maintain close contact with school leadership.
	Management
CiDAME51	Build a motivated team, allow voluntary departures, and recruit selectively.
CiDAME52	Grand autonomy
CiDAME53	Encourage creativity within teams
CiDAME54	Ensure responsiveness of service manager and pedagogical coordinator.
CiDAME55	Hold institutional meetings.
CiDAME56	Protect information-sharing time.
CiDAME57	Allow flexibility to adapt standard procedures.
	Training
CeDAME5	Initial training
CeDAME6	Professionals' previous experience
CiDAME58	Continuous training supported by DAME
	Professionals' dispositions
CeDAME7	Professionals show perseverance, driven by a strong conviction (acting for a 'Cause').
CiDAME59	Evolving missions can affect motivation (focus shift, travel, isolation).

Partners

CeDAME8	Stability of professionals supporting the CYWD, whether external (e.g. therapists), from medico-social structures (SESSAD), or other sectors (child welfare).

68 ingredients linked to National Education (52 CiEN and 16 CeEN)

Ingredients related to the conditions of welcoming the CYWD in the school and in the classroom

<u>Ingredients related to the chosen modalities of schooling for the CYWD</u>

CiEN1 The schooling configuration chosen for the CYWD is appropriate and adapted.

CiEN2 Parents drop off their children at school in the morning.

<u>Ingredients related to the modalities of welcoming the CYWD at school</u>

CiEN3 The CYWD can take part in all class and school activities without logistical 'barriers', as the search for adjustments is prioritized.

CiEN4 The teacher and the school are the main contacts for the CYWD and their family; the DAME does not act as an intermediary.

CiEN5 Difficult situations or conflicts with the school are handled directly by the school.

<u>Ingredients related to the modalities of classroom functioning</u>

CiEN6 The CYWD sits next to another student; this seat is not reserved for the AESH.

CiEN7 The CYWD has the same attributes as the rest of the class.

CiEN8 The presence of the CYWD does not disrupt the class.

CiEN9 The class benefits from the inclusion.

CiEN10 The class functions well in the presence of the CYWD.

CiEN11 In class, DAME professionals and AESH also provide support to other students who need it.

CiEN12 Pedagogical adaptations designed for the CYWD also benefit other students in the class.

CiEN13 The DAME teacher can intervene in co-teaching in the classroom.

CiEN14 Opportunities for interaction between classmates are encouraged and maintained.

CiEN15 The CYWD is included in a collective learning dynamic.

CeEN1 Composition et nombers of the class

<u>Ingredients related to the teacher's intervention with teh CYWD</u>

CiEN16 The teacher dedicates individual time to the CYWD, knows them well, and can track their progress.

CiEN17 The CYWD works, as much as possible, using the same materials as their classmates, with objectives adapted to their needs.

CiEN18 The teacher understands that the learning path of a CYWD may differ greatly from that of another student.

CiEN19 The teacher adapts expectations to meet the student where they are and builds from their abilities, developing an Individualized Learning Program (PAOA).

CiEN20 The teacher invents or adapts solutions for each situation, engaging in a cycle of trial and adjustment.

CiEN21 The teacher identifies even minimal progress by the CYWD and highlights it to the student and the class.

CiEN22 The teacher creates conditions for progress and allows the CYWD to take on small challenges to improve.

CiEN23 The teacher emphasizes the CYWD’s strengths.

CiEN24 The teacher assigns valued roles to the CYWD.

CiEN25 The teacher uses Universal Design for Learning.

CiEN26 The teacher maintains a positive attitude toward the CYWD.

Ingredients linked to the school and the Academy

<u>Learning and training</u>

CiEN27 Learning through experience.
CiEN28 Exchange and peer learning during formal or informal times.
CiEN29 Training on 'School for All' offered to teachers, including Universal Design for Learning.

The management supports through :

CiEN30 School leadership fosters collaboration and support among teachers.
CiEN31 Flexibility in class composition to consider teachers' preferences.
CiEN32 Anticipating the arrival of CYWD at the beginning of the school year.
CiEN33 Using school councils to dedicate time to inclusive practices.
CiEN34 Harmonizing teaching methods so tools designed for CYWD benefit all classes.
CiEN35 Embedding 'School for All' into the school project.
CiEN36 Inscrivant des objectifs quantitatifs de co-intervention dans le projet d'établissement
CiEN37 Mobilizing support networks: RASED, SEI.
CiEN38 Acting as mediator with the Inclusive School Service of the Academy.
CiEN39 Promoting teacher training within the school.

The Inclusive School Service of the Academy

Providing support to specialized teachers.

CiEN40 Monthly meetings for pedagogical coordinators.
CiEN41 Training for specialized teachers
CiEN42 Training for teachers
CiEN43 Training on Universal Design for Learning.
CiEN44 Promoting joint training between DAME professionals and teachers.
CiEN45 Possibility to receive individual follow-up.
CiEN46 Possibility to be consulted.
CiEN47 Flollow up within schools.

Promotes the implementation of the program among schools and teachers.

CiEN48 Close relations with the Academic Director of National Education Services and the National Education Inspector.
CiEN49 Close relations with school management.
CiEN50 Training sessions offered to teachers (in collaboration with district directors).
CiEN51 Possibility of being consulted

Ingredients linked to the national system

Structural factors

CeEN2 The school year is organized into periods.
CeEN3 Preparation time before the school year is insufficient.
CeEN4 No hierarchical link between school management and teachers.
CeEN5 No time allocated for teamwork in teachers’ schedules.

Pedagogical approaches

CeEN6	Possibility to adapt existing school programs through a " Programmation adaptée des objectifs d'apprentissage" (PAOA).
CiEN52	Development of Universal Design for Learning.
Teachers management	
CeEN7	No hierarchical link between school management and teachers.
CeEN8	Assignment and transfer of staff in the national education system (the “mouvement”).
CeEN9	Specialized teachers are sometimes inexperienced and may take these positions by default (less requested positions, "mouvement").
CeEN10	School management does not have the possibility to form their team based on motivation criteria.
CeEN11	Job reference frameworks.
Inclusive school	
CeEN12	Administrative burden linked to the presence of a CYWD in the classroom.
CeEN13	Frequent policy changes and pilot projects may disrupt implementation.
CeEN14	Precarious working conditions for AESH (unfilled posts, high turnover).
CeEN15	Specialized teachers belong to the national education system.
Other programs	
CeEN16	Multiplicity of programs (non-native, gifted, ...) with non-shared staff.

* The "Livret Parcous Inclusif" (iInclusive pathway record) is an application designed to simplify information sharing between the educational team, the family, and the MDPH (since 2023).

5 ingredients linked to the National Education/DAME cooperation

CiCoDE1	A shared educational and pedagogical project that underpins professional practices and helps them evolve collectively: the teacher enriches their practices through interaction with DAME professionals and gradually acquires the appropriate gestures/attitudes for the CYWD in their class; DAME professionals, in addition to supporting CYWD, develop a role of support and resource for teachers.
CiCoDE2	Shared tools to streamline information and decision-making on scheduling / promote teamwork (e.g. crisis management protocol for a CYWD).
CiCoDE3	Teamwork, within and between institutions, must be encouraged — good coordination between teachers and DAME professionals working together as a team.
CiCoDE4	The classroom teacher and the specialized DAME teacher have built a relationship of trust, and the teacher agrees to entrust their class to the DAME teacher.
CiCoDE5	The teacher and the professionals supporting the CYWD are able to respond appropriately in cases of challenging behavior from the CYWD. They revisit the issue with the CYWD in a consistent, stable, and collaborative manner. Faced with this structured framework, the CYWD can potentially make progress, and the class environment is strengthened
